# Successful Foot Salvage in a Patient With Diabetes Following Split-Thickness Skin Grafting and Antidiabetic Therapy: A Case Report

**DOI:** 10.7759/cureus.101889

**Published:** 2026-01-20

**Authors:** Ernest Chukwuma, Ehondor Michael, Ehondor Malcolm, Amietubodie Miebaka, Anita Osawota, Kalu Victor O., Joy Agu, Ginika S Okafor, Ebenezer Tamunoseipiriye, Chinua Onyebuchi

**Affiliations:** 1 Surgery, Castle Hospital, Portharcourt, NGA; 2 Surgery, Federal Teaching Hospital, Ido-Ekiti, NGA; 3 General Practice, Edo State University Teaching Hospital, Benin, NGA; 4 Obstetrics and Gynaecology, Rivers State University Teaching Hospital, Portharcourt, NGA; 5 Obstetrics and Gynaecology, Nisa Premier Hospital, Abuja, NGA; 6 Surgery, University of Portharcourt Teaching Hospital, Portharcourt, NGA; 7 Preventive Medicine, Afe Babalola University, Ekiti, NGA; 8 Internal Medicine, Federal Teaching Hospital, Ido-Ekiti, NGA; 9 Epidemiology and Public Health, Rivers State University Teaching Hospital, Portharcourt, NGA; 10 Neuroendocrine, Full Bar Health Group, Abuja, NGA; 11 General Medicine, Royal College of Physicians of Ireland, Dublin, IRL

**Keywords:** advanced wound care, foot perfusion, incisional negative pressure wound therapy, peri-operative glycemic dynamics, split thickness skin graft

## Abstract

Diabetes mellitus remains a major global health burden, particularly affecting wound healing in patients with traumatic injuries. Chronic hyperglycemia impairs tissue regeneration and immune function, increasing the risk of infection, delayed recovery, and potential limb loss. We aim to demonstrate the critical importance of glycemic control and multidisciplinary management in enhancing wound healing outcomes among diabetic patients undergoing surgical interventions. using the case of a 65-year-old male with a crush injury to the left foot following a road traffic accident. The patient underwent surgical debridement and split-thickness skin grafting. Postoperative complications prompted further clinical evaluation, revealing poorly controlled diabetes. A multidisciplinary team approach was initiated, incorporating endocrinological, surgical, nutritional, and antimicrobial strategies. Initial graft take was poor, accompanied by signs of systemic infection, including fever, leukocytosis, and hyperglycemia. However, following glycemic and nutritional optimization, wound healing significantly improved, and graft uptake was observed within two weeks. The patient was discharged after approximately two months with satisfactory healing, therefore highlighting the indispensable role of tight glycemic control in the postoperative care of diabetic patients. A multidisciplinary, holistic approach integrating endocrine, surgical, antimicrobial, and nutritional support significantly improves healing outcomes and reduces the risk of limb loss. Early metabolic intervention is essential to optimize recovery and enhance quality of life in diabetic wound care.

## Introduction

Diabetic foot ulcers (DFUs) are among the most challenging complications of diabetes mellitus, contributing significantly to patient morbidity and healthcare burden. Poor glycemic control, impaired perfusion, infection, and nutritional deficiencies are well-documented inhibitors of wound healing in this population [1-3]. Among surgical options, split-thickness skin grafts (STSGs) are often employed to achieve wound closure once infection and metabolic derangements are corrected. However, their success is highly contingent upon optimizing systemic conditions, particularly glycemic control [4].

Emerging evidence highlights that multidisciplinary care-including endocrinological, nutritional, infectious disease, and surgical collaboration-can significantly improve healing outcomes in complex diabetic wounds [5]. This case report underscores the pivotal role of coordinated management, emphasizing glycemic optimization and nutritional support, in achieving rapid graft uptake and healing in a patient with poor baseline control and severe foot trauma.

## Case presentation

We present a 65-year-old male, a retired civil servant with tertiary-level education, who resides in Eleme, Rivers State. He hails from Onne, Rivers State, and is a Christian of the Pentecostal denomination.

He was referred to our facility following a road traffic accident, having sustained a crush injury to the left foot 6 hours before presentation.

He was in his usual state of health until six hours before presentation, when he was being transported on a motorcycle when he fell and slid under a heavy-duty truck. The truck, which was on moderate speed, rolled over his foot, leading to the injury. There was associated pain at the affected site, dull in nature, non-radiating, with no relieving or aggravating factors, and of very severe intensity. There was associated bleeding from the injury, estimated at approximately 50 mL, frank red in color, with no passage of clots. There was no bleeding from any other site. There was no loss of consciousness (LOC) or head trauma.

Following the onset of symptoms, he was rushed to a private facility by nearby pedestrians, where he received intramuscular tetanus toxoid, analgesics, and antibiotics. The wound was dressed, and he was subsequently referred to our facility for expert management.

He was not a known hypertensive, diabetic, epileptic, asthmatic, or dyspeptic. His blood group was A⁺, and his genotype was AA.

He had no history of previous surgeries, hospital admissions, or blood transfusions.

He did not smoke or use tobacco products in any form and had no known drug allergies.

On arrival, he was stabilized by the emergency team and subsequently reviewed by the orthopedic team. Initial examination revealed an elderly man in mild painful distress, afebrile, anicteric, acyanosed, and not dehydrated. His vital signs were as follows: respiratory rate 24 cycles per minute (cpm), SpO₂ 96%, pulse rate 74 beats per minute (bpm), blood pressure 155/80 mmHg, and random blood sugar (RBS) 88 mg/dL.

**Figure 1:**
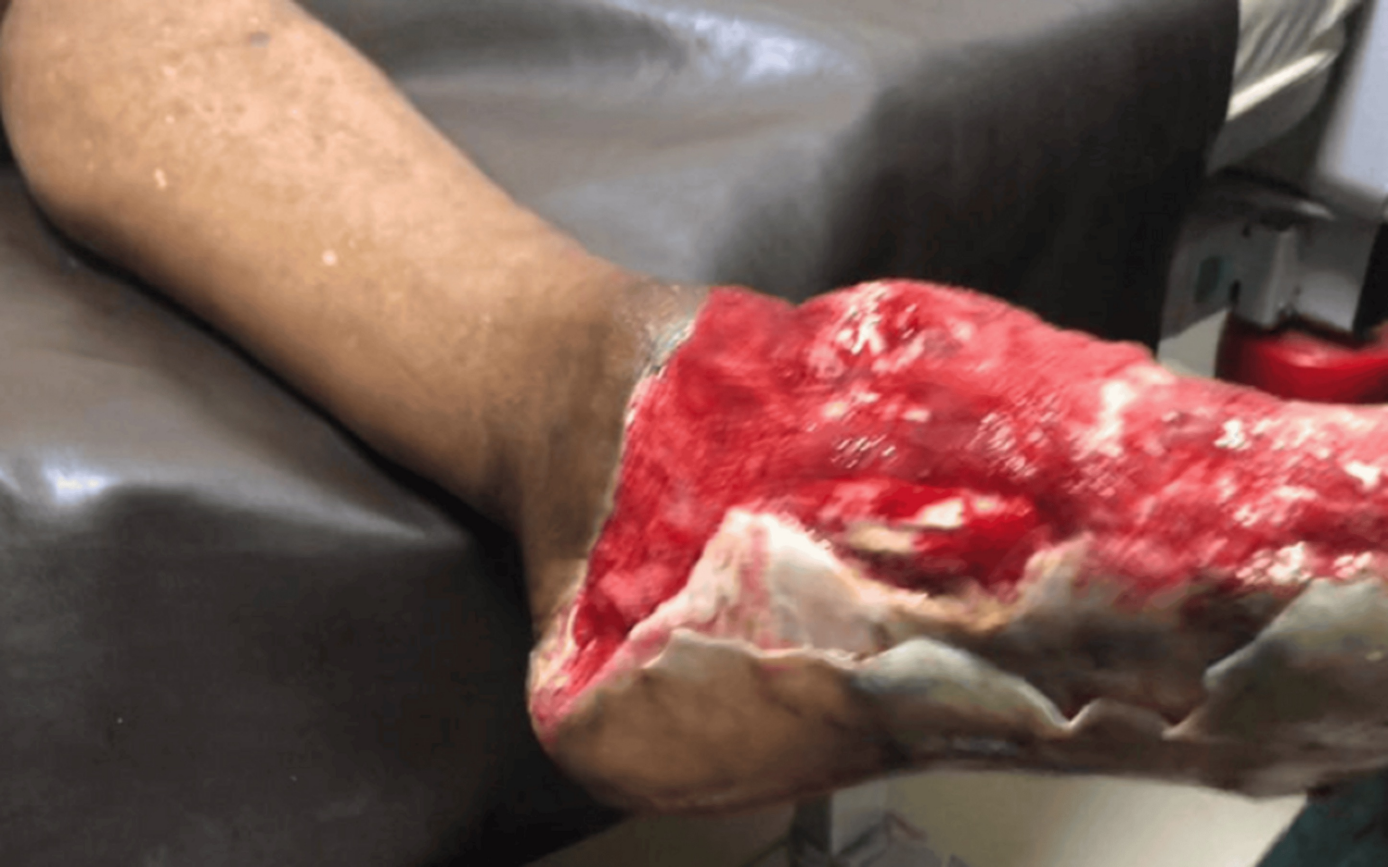
Wound before treatment and grafting (side view).

Musculoskeletal examination showed a dressing soaked with serosanguinous fluid. Upon removal, there was an extensive reddish avulsion wound involving the entire dorsum and medial aspect of the left foot, measuring approximately 8 cm × 6 cm, with associated degloving injury extending into the central plantar region. The first and second toes were traumatically amputated, with exposure of tendons, muscles, and superficial blood vessels, as shown in Figure 1. The foot was non-tender to deep palpation, with warm overlying skin and preserved dorsalis pedis pulse. Ankle movement and toe wiggling were intact.

**Figure 2:**
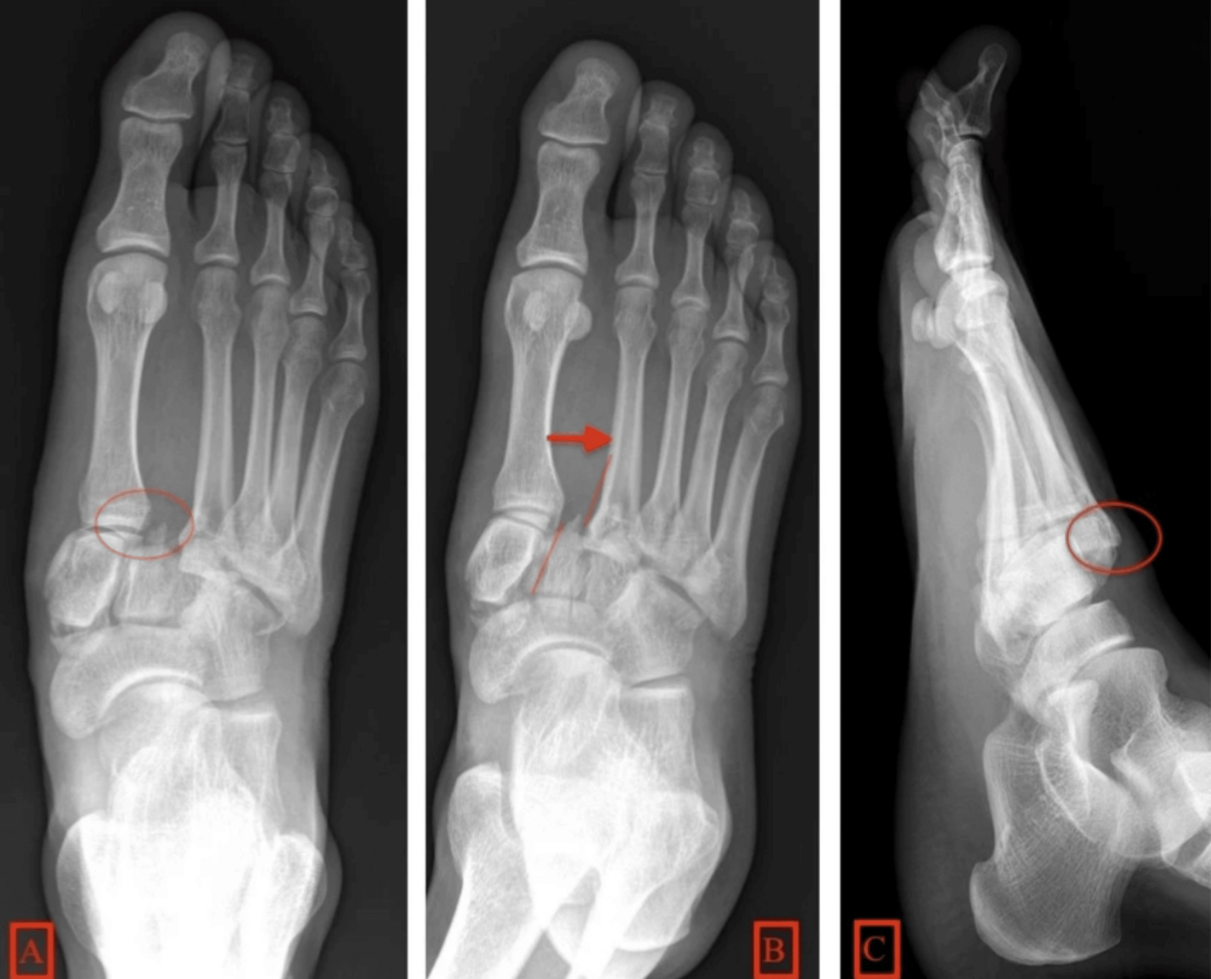
Lisfranc fracture. (A) AP radiograph demonstrating the circled Lisfranc ligament avulsion fracture. (B) The arrow shows the increase in distance between the first and second metatarsals. Red lines show misalignment between the second metatarsal bone and the second cuneiform. There is also a fracture of the first cuneiform seen with a lateral shift of the second, third, fourth, and fifth metatarsals. (C) Lateral radiograph shows dorsal subdislocation of the metatarsal base (red circle).

A plain radiograph of the left foot revealed a fracture of the medial cuneiform and lateral displacement of the base of the second and third metatarsals, consistent with a Lisfranc injury (Figure 2). Initial diagnoses included crush injury with traumatic amputation of the first and second toes and Lisfranc dislocation. Routine blood tests showed a slightly reduced packed cell volume (PCV), normal electrolyte levels and renal function, and negative HIV and hepatitis serology.

The wound was irrigated with 6 L of normal saline and managed with intravenous ceftriaxone (1 g BID) and tinidazole (800 mg daily). Pain was managed with intramuscular pentazocine (30 mg, PRN) and oral nonsteroidal anti-inflammatory drugs (NSAIDs). A tetanus toxoid booster was not administered, as it had been given at the referring center. A posterior back slab was applied, and daily dressings with povidone-iodine were commenced.

On the fourth day, the plastic surgery team subsequently reviewed the patient and confirmed a diagnosis of crush injury to the left foot. A wound swab for microscopy, culture, and sensitivity (MCS) showed no growth. However, based on clinical signs of possible infection and worsening systemic parameters, intravenous cefotaxime 1 g BID was commenced empirically for six days. A fasting blood glucose test was also performed and was found to be elevated. Nutritional support was initiated with oral vitamins A, C, and E. Daily wound care transitioned to hydrogel spray dressings. The patient was optimized and scheduled for delayed surgical intervention.

**Figure 3:**
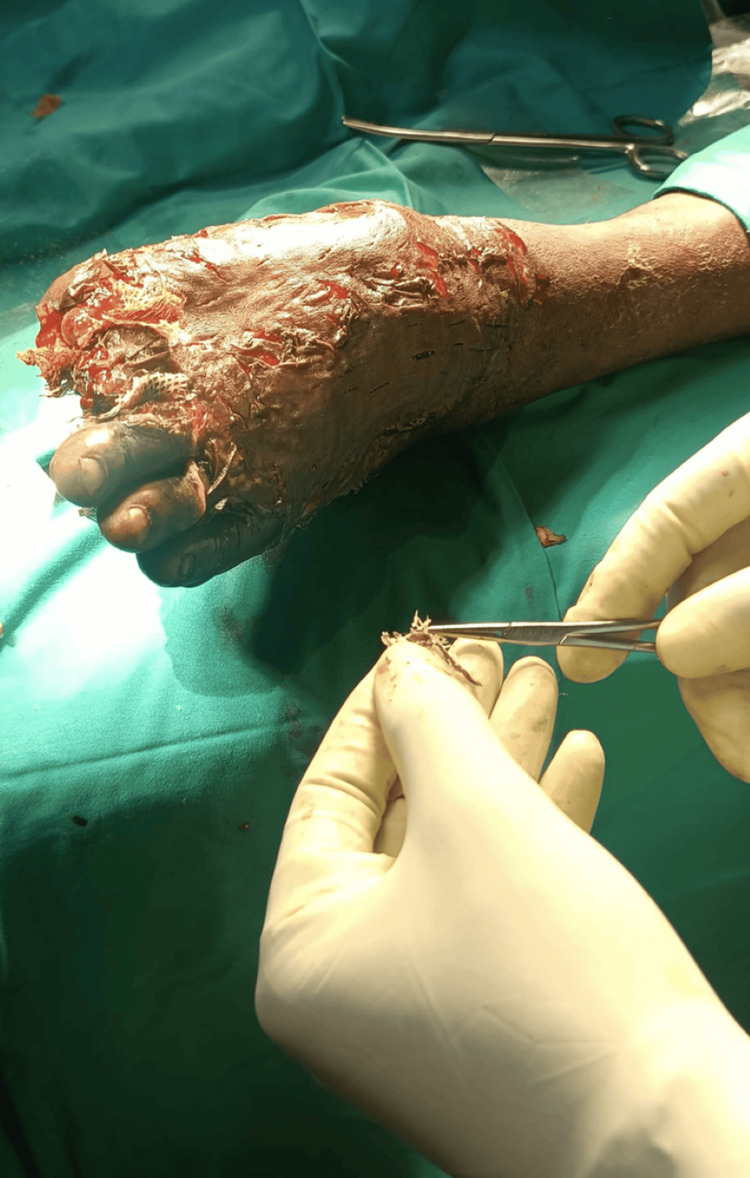
Wound during surgery (split-thickness skin grafting).

**Figure 4:**
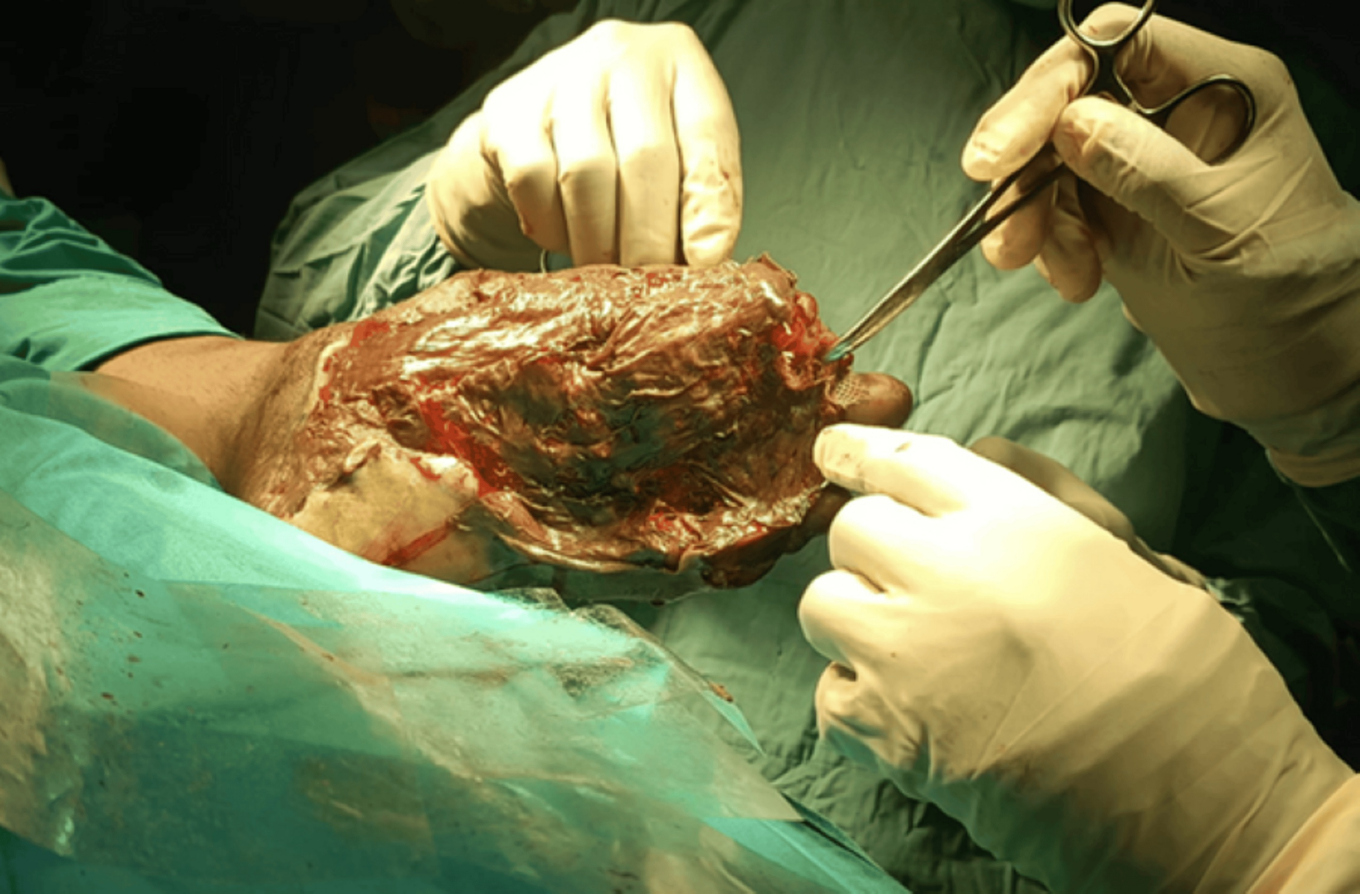
Wound during surgery.

On Day 31, after evidence of healthy granulation tissue, the patient underwent STSG under spinal anesthesia. Intraoperative findings included granulation over the dorsum of the foot, necrosis of the plantar bed, and disarticulation of the fourth and fifth toes. The graft was harvested from a suitable donor site, applied to the debrided wound, and dressed with betadine-soaked gauze and calendula oil. The grafting procedure is shown in Figures 3-4.

On Day 38, the graft appeared nonviable. There was tachycardia of 108 bpm and tachypnea of 31 bpm. An RBS at this point was unrecordably high, and the patient was febrile (38.2 degrees Celsius) and disoriented. Endocrinology was urgently consulted. Laboratory findings showed leukocytosis (elevated WBC; Table 1), and the patient was assessed as having sepsis complicating poor glycemic control. Treatment included subcutaneous insulin (Humulin R) 10 IU TID, intravenous metronidazole 500 mg TID, ceftriaxone 1 g BID, and paracetamol 600 mg TID.

**Table 1:** Clinical timeline of events. - indicates that the test was not done or not recorded. >33.0 indicates a glucose level above the measurable range of the glucometer. PCV, packed cell volume; MCS, microscopy, culture, and sensitivity; BP, blood pressure; STSG, split-thickness skin graft

Day	Clinical event/intervention	FBG (mmol/L)	RBS (mmol/L)	PCV (%)	BP (mmHg)	Wound status	WBC count (x10^9^)
1	Admission: wound irrigation, IV Rocephin, and Tinidazole	-	4.9	32	155/80	Degloving injury with exposed tissue	7.4
3	Plastic surgery review; MCS showed no growth; IV Cefotaxime started	6.2	-	32	121/85	Stable; antibiotics optimized	7.4
7-13	IV Cefotaxime 1 g BID for 6 days	-	-	-	-	Wound granulating	-
31	STSG performed	-	-	31	136/68	Granulation present; graft applied	-
38	Graft nonviable; febrile; glucose unrecordable	-	>33.0	28	155/100	No graft take	25.8
40	Endocrinology review: insulin started	13.5	26.5	28	160/92	Sepsis managed	10.6
46	Antidiabetics revised	23.9	-	28	136/82	Infection resolving	10.6
55	Glycemia normalized; transfused 1 unit of blood	6.7	10.9	24	148/84	The wound began healing	-
59	PCV improved; BP stable	6.5	-	30	Normal	Satisfactory graft uptake	8.5
61	Discharged	-	-	30	Normal	Healing satisfactory	8.5

Reference laboratory values are shown in Table 2.

**Table 2:** Normal laboratory values. The table shows the reference ranges for the quantitative laboratory values presented above, although these may vary between laboratories.

Parameter	Full name	Normal range	Unit	Notes/clinical significance
FBG	Fasting blood glucose	3.9-5.5	mmol/L	Measured after at least 8 hours of fasting; ≥7.0 mmol/L on two occasions suggests diabetes mellitus.
RBG	Random blood glucose	<7.8	mmol/L	Taken any time of the day; ≥11.1 mmol/L with symptoms suggests diabetes mellitus.
PCV	Packed cell volume (hematocrit)	Males: 0.40-0.54; females: 0.36-0.48	L/L or %	Indicates proportion of blood made up of red cells; low in anemia, high in dehydration/polycythemia
WBC	white blood cell count	4.0-11.0 × 10⁹	/L	Elevated in infection/inflammation; decreased in bone marrow suppression or severe infection

**Figure 5:**
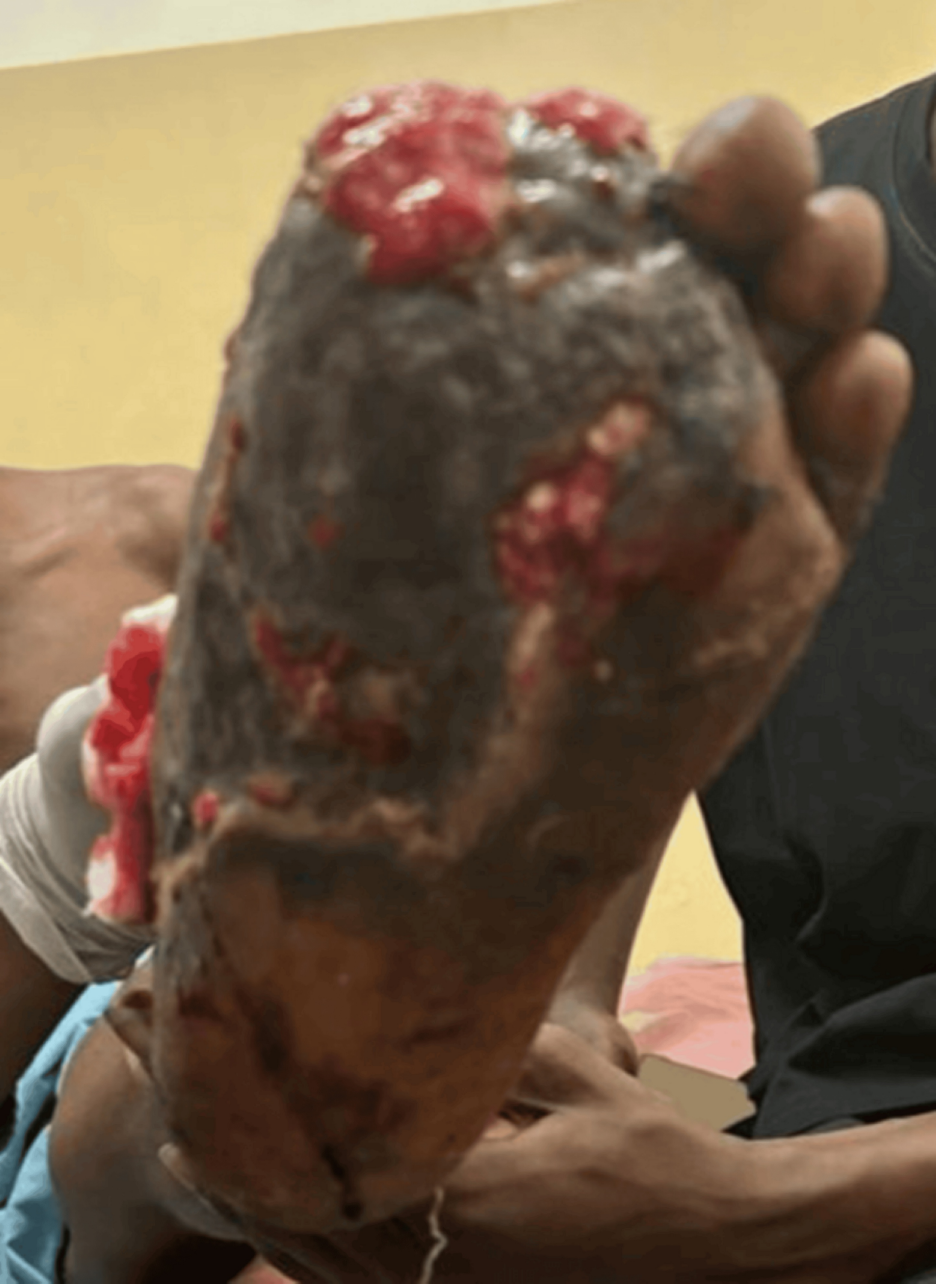
Poorly healing wound after surgery.

On Day 40, fasting blood glucose was markedly elevated, and blood pressure had risen to 160/92 mmHg. After review, the patient was started on Telmisartan 40 mg daily and Nifedipine 30 mg daily. By Day 46, his fasting blood glucose remained elevated despite insulin therapy. His regimen was revised to include metformin 500 mg BID, subcutaneous Lantus 14 IU at night, and short-acting insulin 12 IU TID. The wound is shown in Figure 5.

By Day 55, fasting blood glucose had improved, with visible signs of graft adherence and healing. PCV had decreased, prompting the transfusion of one unit of blood. The PCV results are shown in Table 1. Other lab values were normal, and the patient had regained full orientation. Due to persistent hypertension and anemia, the nutrition and dietetics team was consulted. The patient was counseled on the DASH (Dietary Approaches to Stop Hypertension) diet, advised to restrict salt and fried foods, and encouraged to consume a high-protein diet (e.g., dairy, legumes, boiled eggs), along with hematinic supplementation.

**Figure 6:**
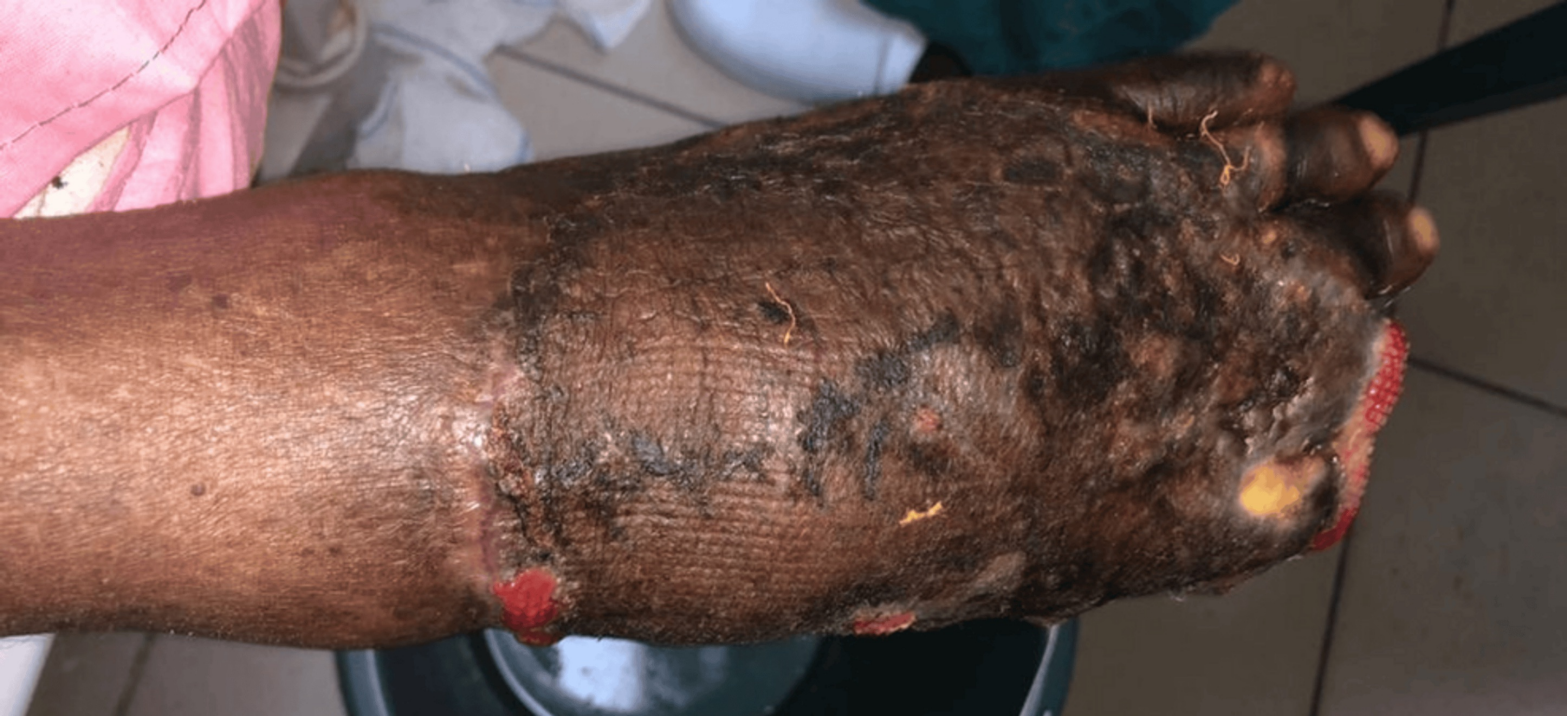
Wound during follow-up showing satisfactory healing.

By Day 59, PCV improved and was normalized, blood pressure stabilized, and the graft site showed satisfactory healing. The patient was discharged on Day 61 and scheduled for follow-up in the plastic surgery and cardiology outpatient clinics. At one-month follow-up, wound healing remained satisfactory, with stable graft uptake, no signs of infection, and intact surrounding skin. The wound is shown in Figure 6.

## Discussion

Wound healing in patients with diabetes is often delayed due to a complex interplay of factors, including chronic hyperglycemia, infection, impaired perfusion, and nutritional deficiencies. This case illustrates the challenges and eventual success of a multidisciplinary approach to wound management and metabolic optimization in a patient with diabetes following foot trauma requiring STSG.

Hyperglycemia is a well-established inhibitor of wound healing. Persistent high glucose levels impair leukocyte function, delay fibroblast proliferation, and reduce collagen deposition, all of which are critical to tissue regeneration [1,2]. In this case, the patient initially presented with normal blood sugar values but subsequently experienced unrecordably high glucose levels postoperatively. This acute glycemic dysregulation likely contributed to the initial failure of graft uptake, consistent with prior findings that link poor glycemic control to graft failure and postoperative infections [3].

Prompt endocrinology involvement and initiation of intensive insulin therapy led to eventual glycemic control, which coincided with visible improvement in graft healing. This underscores the need for early recognition and aggressive management of hyperglycemia in postoperative patients with diabetes. Studies have shown that tight glycemic control (<7 mmol/L fasting) is associated with significantly improved wound closure rates and a reduced risk of infection [4].

Another critical factor in this patient’s recovery was nutritional support. The patient’s anemia and suspected protein deficiency (suggested by poor wound granulation and low PCV) were managed with blood transfusion, hematinics, and a high-protein diet guided by a nutrition team. Malnutrition, especially protein deficiency, has been shown to impair fibroblast activity and angiogenesis, both essential for graft survival [5].

The case also highlights the importance of infection control. Although wound swab cultures showed no growth, clinical signs suggested occult infection, warranting empiric antibiotic coverage. Later, sepsis and leukocytosis required escalation of care, suggesting superinfection that may have further compromised the graft. Infection remains one of the top predictors of STSG failure in diabetic foot wounds [6].

Furthermore, this case exemplifies the benefit of a multidisciplinary approach, combining surgery, endocrinology, infectious disease management, and nutrition. Such integrated care models have been associated with improved amputation-free survival and wound healing outcomes in patients with DFU [7].

Finally, the use of hydrogel dressings played a role in maintaining a moist wound environment, which has been associated with faster epithelization and reduced pain [8]. This aligns with current best practices in managing complex diabetic wounds.

Although this is a single case, it highlights important clinical lessons for team-based wound care. At one-month follow-up, wound healing was satisfactory, and the graft site remained stable with no signs of infection or dehiscence.

Recommendations

Based on the clinical experience and insights gained from the management of diabetic patients undergoing grafting procedures, we propose the following key recommendations aimed at improving outcomes, optimizing resource utilization, and enhancing evidence-based care.

Early Endocrine Evaluation in Diabetic or At-Risk Patients Undergoing Grafting

We strongly recommend the early involvement of endocrinologists in the preoperative and perioperative evaluation of patients with diabetes or those at risk of hyperglycemia, even in the absence of overt hyperglycemia. Subclinical endocrine dysfunctions, such as insulin resistance, autonomic neuropathy, or latent adrenal abnormalities, may impair wound healing and influence postoperative outcomes. Early endocrine input facilitates individualized glycemic targets, optimization of antidiabetic regimens, and identification of metabolic derangements that may otherwise be overlooked. This proactive approach aligns with emerging evidence, suggesting that endocrine optimization before surgical intervention significantly reduces complications and enhances graft integration.

Formalization of Multidisciplinary Wound Care Pathways in Surgical Units Managing Diabetic Limb Trauma

There is an urgent need to formalize structured multidisciplinary care pathways that integrate endocrinology, vascular surgery, plastic surgery, infectious disease, podiatry, and nursing expertise within surgical units managing diabetic limb trauma. Standardized protocols should include early risk assessment tools, coordinated wound debridement timelines, graft viability monitoring, and antimicrobial stewardship. Such collaborative models have been shown to improve limb salvage rates, reduce length of stay, and streamline decision-making. Institutionalizing these pathways will ensure continuity of care, promote accountability, and facilitate training of junior healthcare professionals in evidence-based diabetic wound care.

Further Research Into Biomarkers for Wound Healing Potential

We advocate for increased research investment into the identification and validation of novel biomarkers predictive of wound healing potential. These may include molecular indicators such as tissue oxygenation levels, local or systemic inflammatory cytokine profiles (e.g., IL-6, TNF-α), markers of angiogenesis, and endothelial function. Such biomarkers could serve as early warning signals to stratify patients according to wound healing capacity and identify those who may benefit from adjunctive therapies such as hyperbaric oxygen, negative pressure wound therapy, or early graft revision. The development of point-of-care assays would further enhance clinical applicability, enabling precision medicine approaches in wound management.

Limitations

As a single case report, the findings are not generalizable. The absence of long-term follow-up beyond one month and the lack of standardized wound scoring tools also limit the ability to objectively assess healing progression. Larger cohort studies would be necessary to validate these findings in broader clinical settings.

## Conclusions

This case reinforces the pivotal role of comprehensive metabolic control, particularly glycemic regulation, nutritional optimization, and timely infection management, in enhancing wound healing and graft survival in patients with diabetes with complex foot trauma. The initial failure of graft uptake was not due to surgical technique alone but reflected the adverse impact of uncontrolled hyperglycemia and unaddressed systemic factors. Remarkable clinical improvement was observed only after a coordinated, multidisciplinary intervention addressing endocrine, nutritional, microbiological, and surgical domains.

Although single-case reports have inherent limitations and cannot be broadly generalized, this case underscores several critical lessons. Early involvement of endocrinology and nutrition teams should not be considered adjunctive, but essential in the preoperative and postoperative care of diabetic patients undergoing grafting procedures. Proactive identification and correction of modifiable metabolic derangements may significantly reduce postoperative complications and improve patient outcomes.

Integrating such multidisciplinary, team-based approaches into standard surgical protocols, particularly in settings managing diabetic foot injuries, could help reduce morbidity, improve graft viability, and prevent avoidable limb loss. Future studies and quality improvement initiatives should further explore how early systemic optimization can be operationalized as a core component of diabetic wound care pathways.
